# Rapid and robust generation of long-term self-renewing human neural stem cells with the ability to generate mature astroglia

**DOI:** 10.1038/srep16321

**Published:** 2015-11-06

**Authors:** Thomas Palm, Silvia Bolognin, Johannes Meiser, Sarah Nickels, Claudia Träger, Ralf-Leslie Meilenbrock, Johannes Brockhaus, Miriam Schreitmüller, Markus Missler, Jens Christian Schwamborn

**Affiliations:** 1 Stem Cell Biology and Regeneration Group, Institute of Cell Biology (ZMBE), Westfälische Wilhelms-Universität Münster, 48149 Münster, Germany; 2 Luxembourg Centre for Systems Biomedicine (LCSB), University of Luxembourg, Esch-Belval, Luxembourg; 3 Institute of Anatomy and Molecular Neurobiology, Westfälische-Wilhelms University, Münster, Germany; 4 Cluster of Excellence EXC 1003, Cells in Motion, CiM, Münster, Germany

## Abstract

Induced pluripotent stem cell bear the potential to differentiate into any desired cell type and hold large promise for disease-in-a-dish cell-modeling approaches. With the latest advances in the field of reprogramming technology, the generation of patient-specific cells has become a standard technology. However, directed and homogenous differentiation of human pluripotent stem cells into desired specific cell types remains an experimental challenge. Here, we report the development of a novel hiPSCs-based protocol enabling the generation of expandable homogenous human neural stem cells (hNSCs) that can be maintained under self-renewing conditions over high passage numbers. Our newly generated hNSCs retained differentiation potential as evidenced by the reliable generation of mature astrocytes that display typical properties as glutamate up-take and expression of aquaporin-4. The hNSC-derived astrocytes showed high activity of pyruvate carboxylase as assessed by stable isotope assisted metabolic profiling. Moreover, using a cell transplantation approach, we showed that grafted hNSCs were not only able to survive but also to differentiate into astroglial *in vivo*. Engraftments of pluripotent stem cells derived from somatic cells carry an inherent tumor formation potential. Our results demonstrate that hNSCs with self-renewing and differentiation potential may provide a safer alternative strategy, with promising applications especially for neurodegenerative disorders.

Recent advances in the field of somatic cell reprogramming have enormously furthered the use and optimization of pluripotent stem cells (iPSCs) since the seminal studies by Yamanaka and coworkers^1^,^2^. Despite enormous progresses have been made in the methods for efficiently generating hiPSCs, their directed differentiation into a specific cell type remains technically challenging. This limited ability to generate pure specific cell populations renders the clinical use of pluripotent-derived cells still an obstacle.

The utilization of somatic fate restricted stem cells may be an alternative to achieve faster and more homogenous differentiation into the desired terminally differentiated cell types. It has been shown in mice that primary neural stem cells (NSCs) bear the advantage of being expandable while maintaining their neurogenic and gliogenic differentiation potential^3^,^4^. Therefore, the use of hiPSC derived hNSCs as a source for glia cells and neurons represents a promising strategy^5^. Stable cultures of hNSC may avoid time and cost consuming maintenance of pluripotent stem cells as well as potential immune rejection and teratoma formation^6^.

Particularly interesting is the possibility of generating human astrocytic cultures starting from the same precursor cells used to generate neurons. Traditionally, astrocytes were solely described as supporting elements for neurons. Now, astrocytes are recognized as essential players involved in brain information processing^7^,^8^,^9^,^10^. Alteration of their functions has emerged as a clear factor in disease pathogenesis, especially for neurodegenerative diseases. Additionally, increasing data support the critical role of astroglia in disease progression. As an example, it has been shown that glia cells have a direct, non-cell autonomous effect on motor neuron survival in a mouse model of amyptrophic lateral sclerosis^11^. Despite this growing evidence, astrocytes are less investigated compared to neurons. The lack of an acknowledged and well-characterized set of human astroglial markers and the difficulty to obtain cultures of human astrocytes under defined conditions is partly accounting for that.

Optimizing robust and reproducible protocols for the derivation of multiple cell types is clearly necessary to fully explore the enormous potential offered by iPSC technology. Several human iPSC lines derived from patients suffering from different diseases have been generated, including Parkinson´s disease^12^ (PD), Alzheimer´s disease^13^ and schizophrenia^14^,^15^. Gene-editing approaches have been used to correct genetic mutations on PD patient derived-iPSC, resulting in the successful reversal of pathological phenotypes^12^. As iPSCs are derivable in a patient-specific manner, they are suitable for autologous engraftments^16^ and for personalized disease modeling^17^. iPSCs might also offer a powerful tool in preclinical research to test both toxicity and efficacy of new drug candidates. Basing preclinical tests directly on the target human cells rather than on surrogate cell models, often from other rodents, hold the promise of leading to more efficient drug screening^18^. Notably, in the field of neurodegenerative diseases, hundreds of compounds have been successfully used in animal models to ameliorate induced-neuropathology or cognitive deficits. Yet this has not translated into effective disease-modifying therapies for humans. Obviously, differences between rodent and human physiology impede translation of rodent results to humans^19^. Thus, the possibility of testing drug candidates on patient-specific cells is of fundamental importance for diseases such as neurodegenerative disorders, which only affect humans.

A second application of iPSC is replacement therapies^20^,^21^. The use of iPSCs overcomes the legal/ethical concerns as well as the risks of immune rejection of human embryonic stem cells (hESCs). Despite these advantages, iPSCs, as well as hESCs, have the inherent potential for teratoma formation^6^.

In this study, we describe the robust and rapid generation of hNSCs from hiPSCs. Furthermore, their maintenance and directed differentiation is described. In contrast to most other NSC studies we focus on a detailed characterization of human astroglial differentiation. We put a specific emphasis on the generation of long-term expandable human astrocyte cultures, which can be of use for modeling of disease-specific pathological traits.

## Materials and Methods

### Cell culture

iPSC from 3 individuals were cultured in feeder-free maintenance media (mTeSR, StemCell Technologies) and detached from plates using dispase (1 mg/mL) for 30min at 37 °C with the help of a scraper in case of big colonies. Colonies were collected by sedimentation and resuspended in “EB Medium”: Knockout™ DMEM (Gibco), 20% Knockout™ SR (Gibco), NEAA (Gibco), penicillin/streptomycin (Invitrogen) and ß-mercaptoethanol (Gibco) supplemented with 10 μM SB-431542 (Ascent Scientific), 1 μM dorsomorphin (Tocris), 3 μM CHIR 99021 (Axon Medchem) and 0.5 μM PMA (Alexis). 48 hours later, the medium was replaced by N2B27 medium (DMEM-F12 (Gibco)/Neurobasal (Gibco) 50:50 supplemented with 1:200 N2 supplement (Invitrogen), 1:100 B27 supplement lacking vitamin A (Invitrogen), penicillin/streptomycin and glutamine (Invitrogen) supplemented with 10 μM SB-431542, 1 μM dorsomorphin, 3 μM CHIR 99021 and 0.5 μM PMA. After additional 48 hours, the medium was replaced by N2B27 medium supplemented with 3 μM CHIR 99021, 0.5 μM PMA and 150 μM ascorbic acid (Sigma Aldrich). Two days later, neural tube like structures were disintegrated by pipetting and small pieces were plated on Matrigel (BD Biosciences) coated 12-well plates in N2B27 medium supplemented with 3 μM CHIR 99021, 0.5 μM PMA and 150 μM ascorbic acid. At day 8, the medium was exchanged by N2B27 medium supplemented with 3 μM CHIR 99021, 0.5 μM PMA, 150 μM ascorbic acid and 20 ng/ml basic (b) FGF (Peprotech). At day 12, cells were detached using dispase and cultivated in hNSC Maintenance Medium on Matrigel coated 10cm dishes. “Human NSC Maintenance Medium” was composed by DMEM HAM’s F12 medium (Gibco) supplemented with 40ng/ml EGF (Peprotech), 40 ng/ml bFGF, N2 supplement, B27 supplement (with vitamin A), glutamine, Penicillin/Streptomycin and hLIF (1.5 ng/ml). Human NSCs were splitted (1:2 ratio) at a confluence of 70–80% by using dispase.

At a confluence of 70–80% the hNSC Maintenance Medium was switched to “Neuron differentiation medium” which was composed by N2B27 Medium supplemented with 10 ng/mL BDNF (Peprotech), 10 ng/mL GDNF (Peprotech), 1 ng/mL TGF-β3 (Peprotech), 200 μM ascorbic acid and 500 μM dbcAMP (Sigma Aldrich). Cultures were tested/harvested after 4 weeks of neuronal differentiation. Astrocytic differentiation was induced by switching the hNSC Maintenance Medium to the basic medium (DMEM HAM’s F12 medium, glutamine, Penicillin/Streptomycin) supplemented with 1% FCS (Gibco)^3^,^4^. Finally, multilinear differentiation was achieved by replacing the maintenance medium by the basic medium containing 10% of FCS.

### Immunocytochemistry

For immunohistochemical analysis, cells were fixed with 4% paraformaldehyde (PFA) in 120mM phosphate buffer (PBS), pH 7.4, permeabilized with 0.05% Triton X-100 in PBS, blocked with 10% goat serum in PBS and subjected to immunohistochemistry staining with primary and secondary antibodies diluted in the blocking solution.

For immunolabelling the following antibodies at indicated dilutions were used: anti-Nestin (1:400; BD Bioscience), anti-Sox1 (1:100; R&D Systems), anti-Sox2 (1:200; Abcam), anti-Pax6 (1:200; DSHB), anti-Ki67 (1:200; Vector Labs), anti-TuJ1 (1:400; Covance), anti-Map2 (1:200; Millipore), anti-DCX (1:200; Millipore), anti-GFAP (1:200; Millipore), anti-O4 (1:50, Sigma-Aldrich), anti-GABA (1:200, Abcam), anti-vGlut1 (1:200; Millipore), anti-TH (1:100, Millipore), anti-Synaptophysin (1:100; Millipore), anti-PSD95 (1:200; Invitrogen), anti-vimentin (1:5000; Abcam), anti-S100β (1:1000; Sigma-Aldrich), anti-aquaporin 4 (AQP4, 1:100; Santa Cruz Biotechnology) and anti-excitatory amino acid transporter 2 (EAAT2, 1:100; Santa Cruz Biotechnology), secondary Alexafluorophore-conjugated antibodies (1:1000, Invitrogen). DNA was stained using Hoechst 33258 (1:10000, Invitrogen). All cells expressing a particular marker were counted manually and normalized to the total number of cells.

### Microarray

mRNA was extracted from hiPSCs, hNSCs and derived cells using the RNAeasy kit (Quiagen) following manufacturer’s recommendations. mRNA quantity and purity were determined by using a NanoDrop ND-1000 spectrophotometer (NanoDrop Technologies). Additional quality check was performed by the Agilent Bioanalyzer (Agilent). Gene expression profiles were generated using HumanGene 2.0ST arrays according to manufacturer’s recommendations (Affymetrix).

### Bioinformatics

Correspondence analysis (CoA) from the TIGR MeV (MultiExperimentViewer; http://www.tm4.org/mev/) expression analysis platform was performed on the whole transcriptome established by the HumanGene 2.0ST arrays after RMA normalization proposed by the Affymetrix Expression Console.

GeneOntolgy (GO) analysis was performed on the 1428 transcripts specifically differing hNSCs from parental hiPSCs and from filial hMLDCs by a 2-fold difference. Here, we used the GO–biological process analyzer implemented in the DAVID analysis platform (http://david.abcc.ncifcrf.gov/)^22^. GO terms and associated p-values (0.05 or lower) were then introduced into the REVIGO webserver^23^ to establish network files. These files were than uploaded to the cytoscape software where the final GO-Network was built up (www.cytoscape.org)^24^.

### RT-qPCR

For quantification analysis, total RNA (mRNAs and miRNAs) was extracted from hiPSCs, hNSCs, human neurons (4 weeks of differentiation) and human astrocytes (6weeks of differentiation) by the RNAeasy kit (Quiagen) following manufacturer’s recommendations. Gene expression levels were evaluated by the SYBR-Green Jump Start Taq Ready Mix (Sigma-Aldrich) following manufacturer’s recommendations. Gene-related intensity levels were evaluated upon normalization with *GAPDH* levels. The following primers were used:

*GAPDH*: GTGGACCTGACCTGCCGTCT, GGAGGAGTGGGTGTCGCTGT

*OCT4*: CCTCACTTCACTGCACTGTA, CAGGTTTTCTTTCCCTAGCT

*NANOG*: TGAACCTCAGCTACAAACAG, TGGTGGTAGGAAGAGTAAAG

*SOX2*: CCCAGCAGACTTCACATGT, CCTCCCATTTCCCTCGTTTT

*Ki67*: ATACGTGAACAGGAGCCAG, CCTTGGAATCTTGAGCTTTCTC

*SOX1*: AATTTTATTTTCGGCGTTGC, TGGGCTCTGTCTCTTAAATTTGT

*NESTIN*: CTGGAGCAGGAGAAACAGG, TGGGAGCAAAGATCCAAGAC

*PAX6*: ATGTGTGAGTAAAATTCTGGGCA, GCTTACAACTTCTGGAGTCGCTA

*GFAP*: CTGCTCAATGTCAAGCTGG, AATGGTGATCCGGTTCTCC

*MAP2*: GGAGACAGAGATGAGAATTCCT, GAATTGGCTCTGACCTGGT

*TH*: GTGCTAAACCTGCTCTTCTC, TTCAAACGTCTCAAACACCT

*SLC6A11 (GABA)*: CAACAACTGCTACAGGGAC, GAGAAGATGGCAAACCCAG

*SLC17A7 (Glutamate transporter)*: CCATGACTAAGCACAAGACTC, AGATGACACCTCCATAGTGC.

### Extraction of intracellular metabolites and Gas Chromatography-Mass Spectrometry

Astrocytes and MLD cultures were cultivated for 6 weeks in 12-well plates and washed with 1 ml of 0.9% NaCl and quenched with 0.2 ml −20 °C methanol. After adding an equal volume of 4 °C cold water, cells were collected with a cell scraper and transferred in tubes containing 0.2 ml −20 °C chloroform. The extracts were shaken at 1400 rpm for 20 min at 4 °C (Thermomixer Eppendorf) and centrifuged at 16,000 × g for 5 min at 4 °C. 0.2 ml of the upper aqueous phase was collected in specific glass vials with micro inserts and evaporated under vacuum at −4 °C using a refrigerated CentriVap Concentrator (Labconco).

Metabolite derivatization was performed using a Gerstel MPS. Dried polar metabolites were dissolved in 15 μl of 2% methoxyamine hydrochloride in pyridine at 40 °C under shaking. After 60 min an equal volume of MTBSTFA was added and held for 60 min at 40 °C. 1 μl sample was injected into an SSL injector at 270 °C in splitless mode. GC/MS analysis was performed using an Agilent 7890A GC equipped with a 30 m DB-35MS + 5m Duraguard capillary column. Helium was used as carrier gas at a flow rate of 1.5 ml/min. The GC oven temperature was held at 100 °C for 2 min and increased to 300 °C at 10 °C/min. After 3 min, the temperature was increased to 325 °C. The GC was connected to an Agilent 5975C inert XL MSD, operating under electron ionization at 70 eV. The MS source was held at 230 °C and the quadrupole at 150 °C. The detector was operated in scan mode with mass range m/z 70–800. The total run time of one sample was 25.00 min. All GC/MS chromatograms were processed by using MetaboliteDetector^25^. Mass isotopomer distributions (MIDs) were determined and corrected for natural isotope abundance using MetaboliteDetector (Hiller *et al.*, 2009). For determination of MIDs the following ions were selected (Wegner *et al.*, 2014): Asparatate_3TBDMS: 418–427; Citrate_4TBDMS: 591–601; Glutamate_3TBDMS: 432–442; Serine_3TBDMS: 390–397; Glycine_2TBDMS: 246–252; Lactate_2TBDMS:261–269.

### Electrophysiology

For electrophysiological recordings coverslips with hNSC derived neurons were transferred to a recording chamber mounted on an upright microscope (Zeiss, Oberkochen, Germany) and kept in a bath solution containing (in mM): NaCl 130, KCl 3, NaHCO3 10, CaCl2 1.5, MgCl2 1, Glucose 11, HEPES 10, pH 7.3 with NaOH. Patch pipettes with 2−4 M were filled with (in mM) K-gluconate 125, KCl 20, EGTA 0.5, MgATP 4, MgCl2 4, Na2GTP 0.3, HEPES 10, pH 7.4 with KOH. Measurements were done with an EPC10 amplifier and Patchmaster software (HEKA, Lambrecht, Germany). For puff-application of KCl or glutamate, a pipette with 15 μm tip diameter was placed 60–100 μm away from the recorded cell. Pressure ejection was controlled by a pico pump (PV830, WPI, Sarasota, FL).

### Measurement of Glutamate Uptake

Astrocytes DIV 80–90 and HEK 293 cells were incubated for 10 minutes at 37 °C in basic media containing 50 M of L-glutamic acid (Sigma). Glutamate uptake was measured using a colorimetric kit (abcam) according to manufacturer’s instructions. Absorbance measurements were normalized to total protein per culture well.

### Mice

12 week old male NOD/SCID mice were kept under standard conditions according to governmental rules and regulations. All experiments have been conducted according to the German Animal Welfare Act and have been approved by the responsible authorities (Landesamt für Natur, Umwelt und Verbraucherschutz Nordrhein-Westfalen). For each transplantation approach, 3 animals were used.

### hNSC transplantation

For stereotactic injections, mice were under deep anesthesia (intraperitoneal injection of 17 μl of 2.5% Avertin per gram of body weight) and fixed into a stereotactic frame (Kopf). Three microlitres of cell suspension (in total: 1 × 10^5^–2 × 10^5^ cells) were injected into the lateral ventricle over 5 minutes using a Hamilton 7005KH 5μl syringe. Following stereotactic coordinates in relation to bregma were used: anteroposterior: 1.4 mm, mediolateral: ±0.84 mm, dorsoventral: −2.5 below skull. In order to control the hNSC fate after transplantation, we introduced an *in vitro* pre-differentiation step in our experimental set-up. At the first step, passage 6 or higher hNSCs were splitted in a 1:2 ratio. Three days later, the hNSC maintenance medium in one of the dishes was changed to neuronal differentiation medium, while the second dish was subjected to astroglial differentiation by replacing the maintenance medium by glial differentiation medium. After one week of pre-differentiation, 1 × 10^6^ to 2 × 10^6^ cells were transplanted into the brain of adult NOD/SCID mice. The cell fate was analyzed 6 additional weeks later.

### Perfusion, sectioning and immunohistochemistry

Mice under deep anesthesia were perfused with 50 ml PBS following 50 ml 4% PFA/1 PBS solution. After dissection, isolated brains were post-fixed in 4% PFA/1 PBS solution over night at 4 °C. 40 μm sagittal brain sections were cut using a Vibratom (Leica VT 1200 S). Free-floating sections were permeabilized in Tris-buffered saline solution with 0.1M Tris, 150mM NaCl, pH 7.4/0.5% Triton-X 100/0.1% Na-Azide/0.1% Na-Citrate/5% normal goat serum (TBS+/+/+) for at least 1 h. The primary antibodies anti-Hu Nuclei (1:200; Millipore), anti-DCX (1:400; Abcam), anti-TuJ1 (1:600; Covance) and anti-GFAP (1:100; Millipore) were diluted in TBS+/+/+ and incubated for 48 h on a shaker at 4 °C. For immunofluorescence staining, secondary Alexa-fluorophore conjugated antibodies (Invitrogen) and Hoechst 33258 (1:10000, Invitrogen) were used. Sections were analyzed with a Zeiss LSM 710 confocal microscope.

### Statistical analysis

Data presented are means ± SEM. Statistical significance was tested with Sigma Plot software. Results were denoted statistically significant when *p* values were < 0.05; number (n) of samples/repeats are given in the Results and Figure legends.

## Results

### Newly generated hNSCs conserved self-renewing characteristics

**Figure 1:**
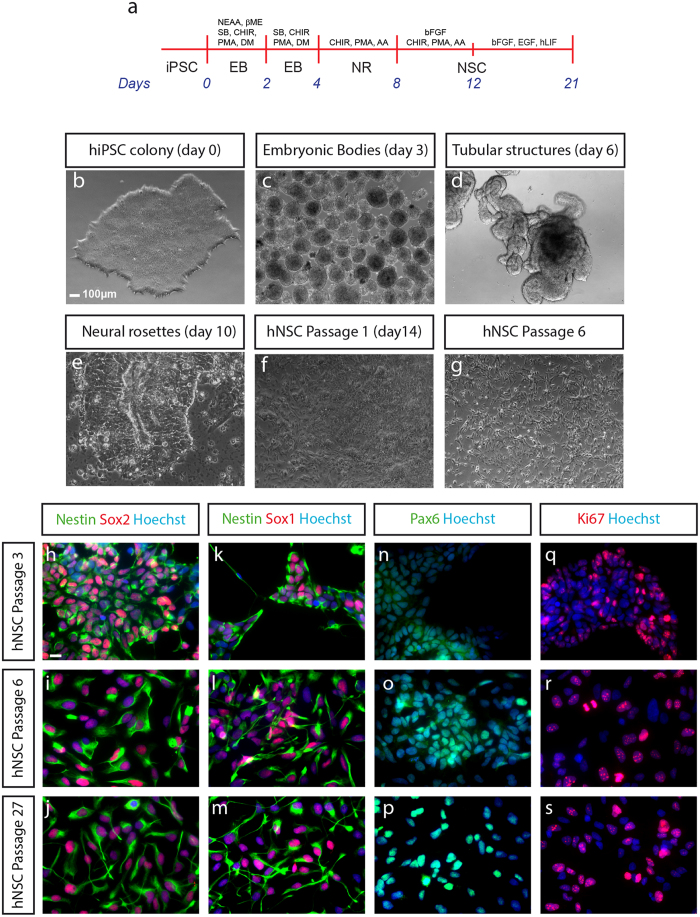
Generation of human neural stem cells (**a**) Schematic representation for directed differentiation of iPSC cells to hNSC. (**b–g**) Phase contrast of images of the generation of hNSC. (**b**) Feeder-free hiPSC. (**c**) Embryonic bodies after 3 days of differentiation. (**d**) Tube like structures after six days of differentiation. (**e**) Induction of neural rosette formation at day 10. (**f**) First passage of hNSCs at day 14 of hiPSC differentiation; (**g**) heterogeneous cell population becomes homogeneous after several passages. hNSC. (**h–s**) Immunofluorescence labeling of the neural stem cell markers Nestin, Sox2, Sox1 and Pax6 as well as of the cell cycle marker Ki67 of hNSCs at Passage 3 (**h**,**k**,**n**,**q**), Passage 6 (**i**,**l**,**o**,**r**) and Passage 27 (**j**,**m**,**p**,**s**). Scale bar 10 μm.

We maintained human iPSCs on mouse embryonic fibroblasts (MEFs) or under feeder free conditions, and treated them according to the scheme in Fig. 1a. As described previously^12^, the neural induction of embryonic bodies from hiPSC (Fig. 1b,c) was achieved by inhibition of BMP and TGFß signaling^26^,^27^. Simultaneously, we administered CHIR99021 and Purmorphamine to stimulate the canonical WNT- and SHH-pathways^28^,^29^. These neural-induced embryonic bodies (Fig. 1c) were cultivated under defined conditions until neural tube like structures appear (Fig. 1d). Neural rosette-like structure formation was induced by supplementing the culture medium with bFGF^3^ for four additional days (Fig. 1e). After re-plating, the cells were cultured in presence of EGF, bFGF, N2, B27 and hLIF. Following the first passages, the initially heterogeneous cell clusters adopted a homogeneous morphology (Fig. 1f,g). Induction of differentiation into either the neuronal or the glial lineage (details see below) induced further changes in morphology.

One key characteristic of neural stem cells is their extensive self-renewal potential. This ability was evaluated by measuring the cell number over the first 21 passages following their generation. The resulting exponential growth curve showed stable proliferation rates over the 21 passages analyzed (Figure S2a). To confirm that generated hNSCs preserved self-renewing characteristics, we evaluated the presence of the stem cell markers Nestin, Sox2, Sox1 and Pax6 at early (passage 3 and 6) and late (passage 27) passages (Fig. 1h–p). While Nestin, Sox1 and Sox2 showed very similar expression patterns, Pax6 displayed cytoplasmic labeling at lower passage numbers and more nuclear labeling at higher passages (Fig. 1n–p), which is in agreement with data on brain development^30^. Finally, we observed that hNSCs maintained proliferation characteristics, as demonstrated by the positive labeling of the cell cycle marker Ki67 across passages (Fig. 1q–s). These results demonstrate that generation and maintenance of hiPSC-derived hNSCs was achieved robustly, and that hNSCs maintained self-renewing characteristics over numerous passages.

### hNSCs revealed distinct gene expression profile

**Figure 2:**
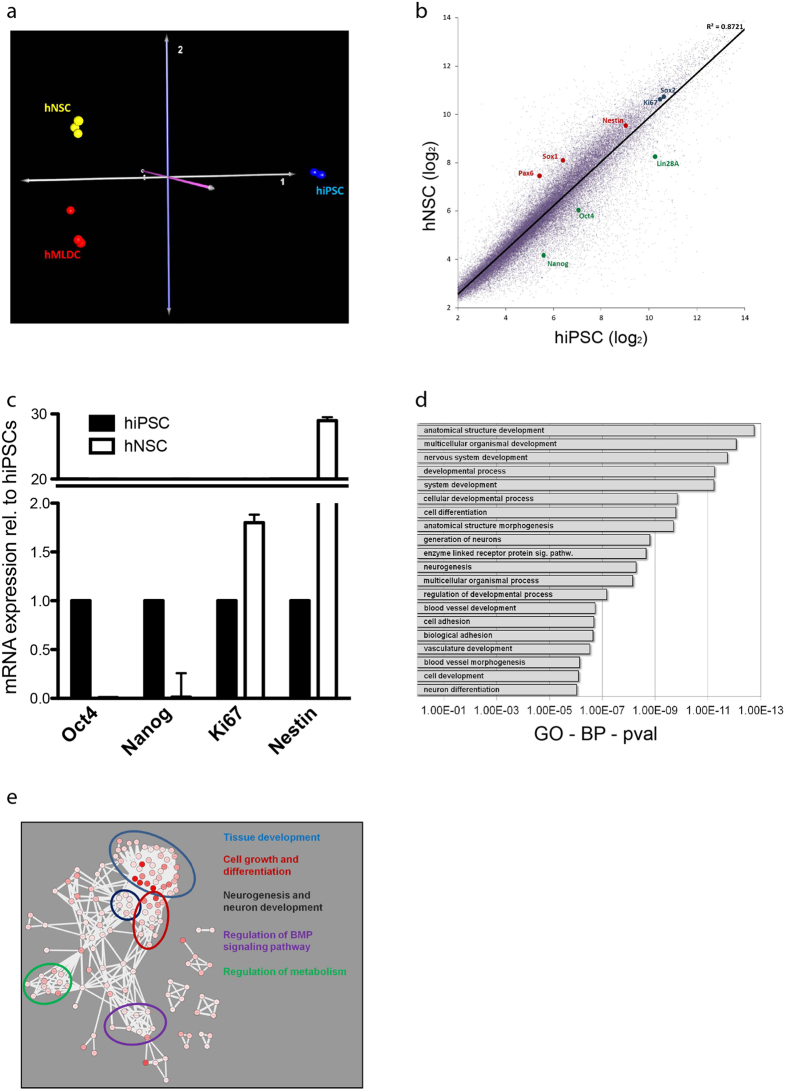
Molecular Characterization of hNSCs (**a**) Multi-Dimensional Scale analysis based on the mRNA transcription profiles of hiPSCs (blue), hNSCs (yellow) and hMLDCs (red). All profiled cell-types were generated from the same individual. (**b**) mRNA scatter blot of hiPSCs and hNSC; hNSC specific genes (red), hiPSC specific genes (green), genes observed in both cell types (black). (**c**) mRNA expression levels of OCT4, Nanog, Ki67, SOX2 and Nestin in hiPSC and hNSC quantified by RT-qPCR. (**d**) Gene Ontology analysis based on the 1428 genes specifically expressed in hNSCs when compared to hiPSCs and hMLDCs. (**e**) GO terms were subjected to a network analysis based on ReviGO (http://revigo.irb.hr/) and CytoScape (http://www.cytoscape.org/) softwares. The main GO sub-clusters are highlighted.

In order to further characterize the generated hNSCs, we compared the mRNA transcriptome of hiPSCs, and hNSCs derived from these progenitors (Fig. 2). Additionally, we included differentiated cells, derived from these hNSCs in the analysis. Since the induced differentiation was undirected, multiple cell types were present in this population; consequently we consider this a multilineage differentiation (hMLDCs) (Figure S1). To minimize the interlineage differences existing between iPSC-lines generated from different individuals^31^, we based our comparison on hiPSCs, hNSCs and hMLDCs derived from the same individual. The transcriptome-based correspondence analysis (CoA) of the three cell types demonstrated that hiPSCs, hNSCs and hMLDCs were characterized by distinct molecular expression signatures (Fig. 2a).

The paired comparison of hiPSCs and of hNSCs in a scatter blot highlights that neural-stem-cell-genes (Pax6, Sox1 and Nestin) were higher expressed in hNSCs when compared to their parental hiPSCs. Similarly, hNSCs showed a significant downregulation of the pluripotency genes Lin28a, Nanog and Oct4 (Fig. 2b and Figure S2b). RT-qPCR analysis comparing the expression levels of the pluripotency genes Oct4 and Nanog as well as the neural-stem-cell-gene Nestin between hiPSCs and hNSCs confirmed these data (Fig. 2c). Interestingly, the neural stem cell specific marker Nestin was found nearly absent in hiPSCs when compared to hNSCs. The marker Sox2, predicted to be strongly expressed within pluripotent^1^ and neural stem cells^32^, as well as the proliferation marker Ki67, exhibited similar expression levels within hiPSCs and hNSCs.

We further isolated 1428 transcripts that differed at least twofold between hNSCs, hiPSCs and hMLDCs. The resulting Gene Ontology (GO) analysis revealed that genes specifically expressed in hNSCs were mainly involved in the biological processes development, differentiation, neurogenesis and cell adhesion (Fig. 2d). In order to better decipher key mechanisms important for hNSC maintenance, we performed a network analysis on the “biological-process GO-terms” that were statistically associated with the 1428 enriched hNSCs genes. Within the GO-Network, we observed that the GO terms clustered as sub-networks that may be outlined as “Tissue development”, “Cell growth and differentiation”, “Neurogenesis and neuron development”, “Regulation of catabolism and metabolism” and “Regulation of BMP signaling pathway” (Fig. 2e). We also compared the transcriptional profiles of hiPSCs, hNSCs and hMLDCs and identified the genes differentially regulated in the 3 groups (Table S1 and Figure S3). By this approach, we were able to define a unique expression signature that clearly distinguished hNSCs from less differentiated hiPSCs as well as from the more differentiated hMLDCs.

### hNSCs differentiated into mature astrocytes

In the next step we wanted to demonstrate the ability of hNSCs to differentiate into astrocytes. The astrocytic differentiation medium consisted of the basic cultivation medium supplemented with 1%FCS. The most commonly used marker protein for astrocytic differentiation is GFAP. However, astrocytes positive for GFAP are considered to show a reactive phenotype while astrocytes negative for GFAP show a quiescent phenotype with protoplasmic morphology, as described previously^33^. We therefore used the additional markers S100β and vimentin to investigate astrocyte differentiation.

**Figure 3:**
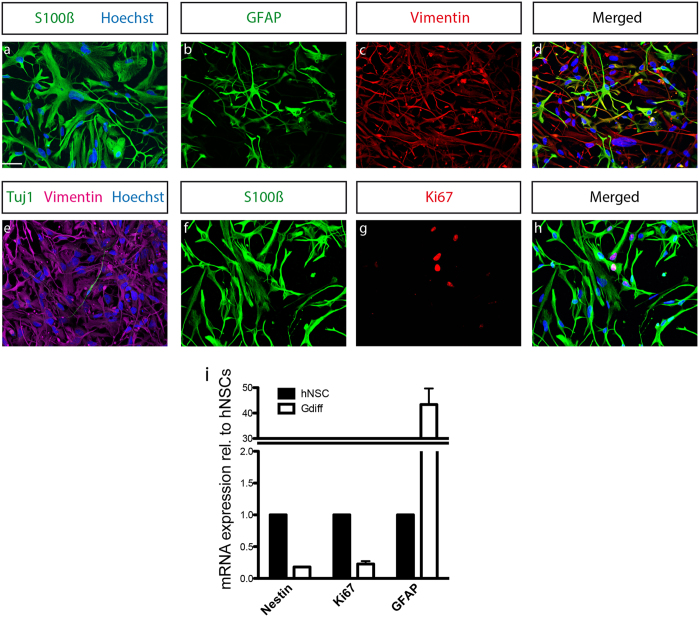
Rapid generation of hNSC derived astrocytes. Representative confocal images illustrating the expression of astrocytic markers (**a**) S100β after 50 days of differentiation, (**b**–**d**) GFAP-vimentin, (**e**) Tuj1-vimentin, (**f**–**h**) S100β-Ki67. (**i**) mRNA expression levels of *Nestin, Ki67, GFAP* in hNSC and astrocyte quantified by RT-qPCR.

After 45–50 days of differentiation, we observed that nearly 100% of the cells were expressing S100β (Fig. 3a). After 60 days, some astrocytes showed GFAP immunoreactivity while all the cells expressed vimentin (Fig. 3b–d). S100ß was expressed in 100% of the cells also at this time point (Fig. 3f–h). The contamination with neurons was negligible as shown by the Tuj1 staining (Fig. 3e). We further observed that after 60 days of differentiation, some cells still expressed the proliferation marker Ki67 (Fig. 3g), indicating that they are able to proliferate, a capacity shared with primary astrocytes^34^,^35^. In support, loss of neural stem cell- and proliferation markers with simultaneous increase of glial markers was also observed by RT-qPCR. *Nestin* and *Ki67* were significantly downregulated upon 45 days of glial differentiation and we observed a massive increase in the expression levels of *GFAP* gene (Fig. 3i).

**Figure 4:**
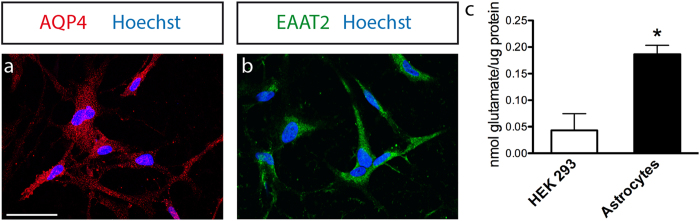
Astrocytes displayed mature functions. Representative confocal images illustrating the expression of astrocytic markers (**a**) AQP4 and (**b**) EAAT2 after 60 days of differentiation. Scale bars 50 μm. (**c**) Glutamate up-take in astrocytes DIV 80–90 and HEK 293. Mean ± SEM; n = 3 indipendent experiments.

Remarkably, after 60 days of differentiation all astrocytes also expressed a basal level of AQP4 (Fig. 4a), a marker for the main water channel in the perivascular membranes, typically present in astrocytic endfeet in brain tissue. Additionally, they expressed the glutamate transporter EAAT2 (Fig. 4b) that is used by astrocytes *in situ* to remove glutamate from the extracellular space. This is a key function of mature astrocytes^36^ that was also confirmed by a glutamate uptake assay (Fig. 4c). Astrocytes differentiated for 80–90 days were able to transport glutamate intracellularly. We assessed HEK 293 as a cell type with no specific glutamate intake ability (p = 0,016, Student *t* test).

Our results demonstrate that by replacing the hNSC maintenance medium with the adequate differentiation medium it was possible to induce a robust and homogenous differentiation into astrocytes. The homogeneous immunoreactivity toward AQP4 and EAAT2 and the ability to transport glutamate suggest that hNSC-derived astrocytes reached a certain level of maturity and functionality.

### Astrocytes showed increased pyruvate carboxylase activity and reduced serine biosynthesis

**Figure 5:**
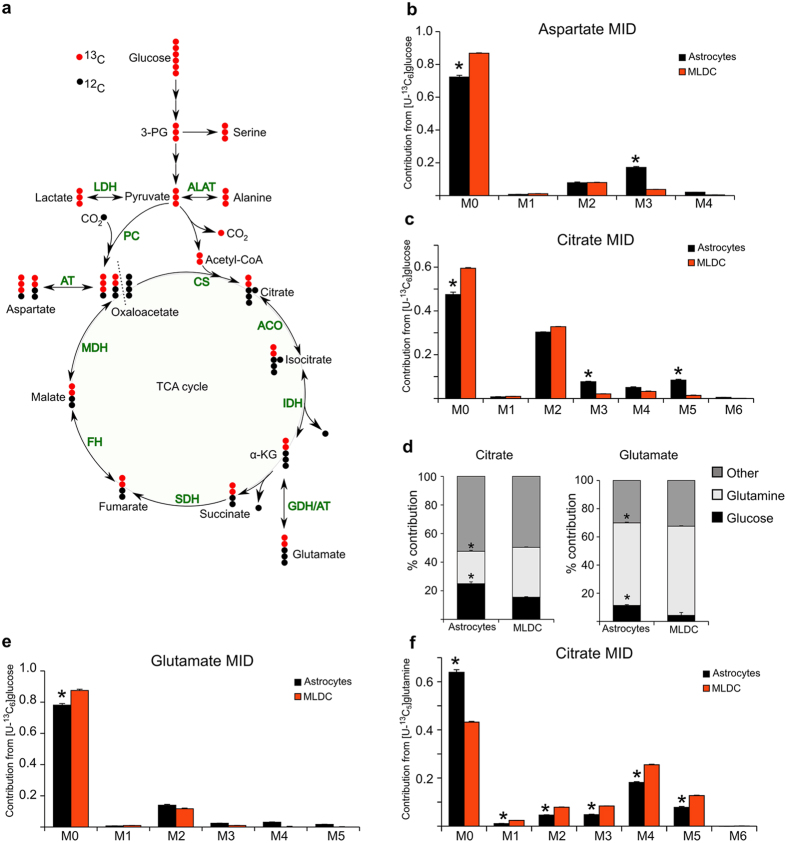
Stable isotope assisted metabolic profiling of hNSCs derived astrocyte. (**a**) Atom transition model of uniformly labeled [U-^13^C]glucose. Glucose derived [U-^13^C]pyruvate can enter the TCA cycle via pyruvate dehydrogenase (PDH) dependent oxidation of pyruvate to acetyl-coA. In this case citrate molecules show a mass increase by two (M2 isotopologue). Subsequent TCA cycle metabolites are also M2 isotopologues. As an alternative way to enter the TCA cycle pyruvate can be carboxylated by pyruvate carboxylase, resulting in oxaloacetate M3 isotopologues. The dotted line indicates the start and the end of the cycle. ALAT: alanine aminotransferase; LDH: lactate dehydrogenase; PC: pyruvate carboxylase; CS: citrate synthase; ACO: Aconitase; IDH: isocitrate dehydrogenase; GDH: glutamate dehydrogenase; AT: aminotransferase; SDH: succinate dehydrogenase; FH: fumarate hydratase; MDH: malate dehydrogenase. (**b–f**) Mass isotopomer distributions (MIDs) and carbon contributions. Cells were labeled for 24h prior extraction of intracellular metabolites and analysis by GC/MS. M1-M6 indicates the number of ^13^C atoms incorporated into the metabolite. (**b**) MID of aspartate using [U-^13^C]glucose as a tracer. (**c**) MID of citrate using [U-^13^C]glucose as a tracer. (**d**) Calculated carbon contribution from gluocose, glutamine and other carbon sources (e.g. lipids, branched chain amino acids) to citrate (left) and glutamate (right). (**e**) MID of glutamate using [U-^13^C]gl = 3 independent experiments in triplicate; *p-*value < 0.05.

A hallmark of astrocytes is the presence and the relative high activity of the metabolic enzyme pyruvate carboxylase (PC)^37^. PC catalyzes the carboxylation of pyruvate to the tricarboxylic acid cycle (TCA) intermediate oxaloacetate. This reaction is important during anabolic processes to replenish the TCA cycle with oxaloacetate. To investigate the presence and the activity of pyruvate carboxylase in hNSCs derived astrocytes we used uniformly labeled [U-^13^C]glucose to monitor the fate of glucose in astrocytes. Application of this stable isotope tracer results in isotopic enrichment in all metabolites downstream of the tracer (*e.g.* metabolites of the glycolysis and the TCA cycle). The isotopic enrichment can be measured using Gas Chromatography/Mass Spectrometry. In case pyruvate is oxidized via pyruvate dehydrogenase to acetyl-coA the succeeding citrate molecule shows a mass enrichment by two (M2 isotopologue). In case of pyruvate carboxylation by PC the succeeding citrate molecule shows a mass enrichment by three (M3 isotopologue). M5 citrate isotopologues result from both, pyruvate dehydrogenase and PC activity (Fig. 5a).

Using this approach, we compared pure astrocyte cultures with multi-lineage differentiation cultures (MLDCs) and we observed increased PC activity in the pure astrocyte culture (17% enrichment vs. 4% enrichment of M3 aspartate) (Fig. 5b). We also observed increased M3 and M5 isotopologue abundance of citrate originating from M3 oxaloacetate as the precursor (Fig. 5c). To compare the amount of glucose contributing to the citrate pool, we calculated the total carbon contribution from glucose to citrate in both culture conditions (Fig. 5d). We determined 24% glucose carbon contribution to citrate in pure astrocyte cultures and only 15% glucose carbon contribution in MLDCs. This difference was also visible in glutamate (Fig. 5d,e). In summary, these data show increased PC activity and glucose derived carbon contribution into the TCA cycle in astrocytes compared to MLDCs.

Besides glucose, the amino acid glutamine represents the most important carbon source for cells. This is especially pronounced in proliferating cells or in any cell type with increased anabolic demands^38^. To evaluate the impact of glutamine to the TCA cycle of astrocytes we used uniformly labeled [U-^13^C]glutamine and investigated the isotopic enrichment in citrate. In the astrocyte cultures we found that glutamine provided similar amounts of carbons for the synthesis of citrate as glucose (22%) (Fig. 5d). However, in MLDCs (where the glucose contribution was lower as in astrocytes) we found an increased contribution from glutamine (34%), probably to compensate for the decreased glucose contribution. In line with increased glutamine derived carbon contribution we also found higher M4 and M5 istopologue abundances of citrate (Fig. 5f).

**Figure 6:**
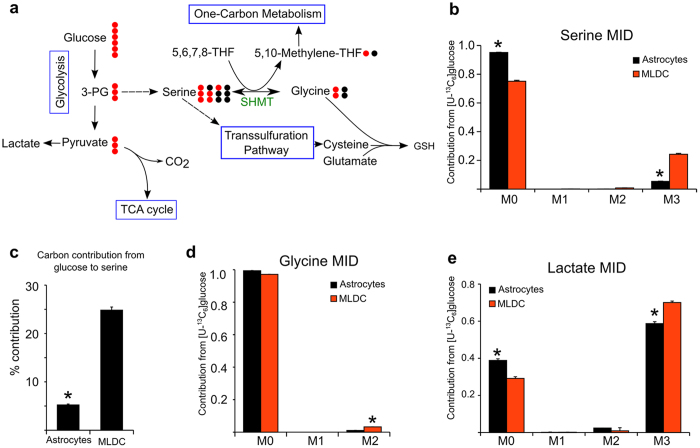
Stable isotope assisted metabolic profiling reveals differences in serine biosynthesis in hNSCs derived astrocytes and hNSCs derived MLDC. (**a**) Cartoon illustrating possible atom transitions for serine in respect to the reversibility of the enzyme serine hydroxy methyl transferase (SHMT) and potential metabolic fates for serine. The hydroxymethylgroup of serine is transferred on tetrahydrofolate resulting in M2 glycine isotopologues. In the reversible reaction serine can be M0, M1, M2 or again M3 depending on the combination of labeled and unlabeled metabolite. (**b**) MID of serine using [U-^13^C]glucose as a tracer. (**c**) Carbon contribution of glucose to serine. Glutamine has no contribution to the serine pool (data not shown) (**d**) MID of glycine using [U-^13^C]glucose as a tracer. (**e**) MID of lactate using [U-^13^C]glucose as a tracer. Mean ±SEM, n = 3 independent experiments in triplicate; *p-*value < 0.05.

Furthermore we identified also unexpected features of astrocyte metabolism compared to MLDCs. By using [U-^13^C]glucose we found clear differences in the MID of serine. This non-essential amino acid can be derived from 3-phosphoglycerate, an intermediate of the glycolysis (Fig. 6a). As an alternative to *de novo* biosynthesis it can be taken up from the medium. Based on stable isotope experiments we found higher relative serine biosynthesis rates in MLDCs (24%) as in pure astrocyte cultures (5%) (Fig. 6b,c) where most serine originated from the medium (M0 isotopologue abundance). Interestingly, although we found at least 5% (astrocytes) and 24% (MLDCs) of relative glucose contribution to serine, there was very little labeling in glycine (Fig. 6d). This indicated that the equilibrium of the SHMT catalyzed reaction is far on the side of serine and that most glycine is taken up from the medium rather than being synthesized from glucose.

Similar to serine, we also found increased labeling in lactate (M3) in MLDCs (Fig. 6e). Lactate has an important role in maintaing the cytosolic redox balance of NADH/NAD^+^. The reason for different lactate labeling patterns could be caused by two reasons: first, pure astrocyte cultures might consume more (unlabeled) pyruvate from the medium (present at 1mM concentration) resulting in higher M0 abundance and second, pyruvate and thus lactate might be produced from serine *via* serine dehydratase. Whether this function plays a role in glial metabolism needs further investigation.

### hNSCs differentiated into various neuronal subtypes

Although we here focus on astrocyte differentiation, the ability to differentiate into neurons is a hallmark of NSCs and therefore needs to be evaluated for the characterization. To induce neuronal differentiation in our hNSC culture system a treatment with the factors BDNF (brain-derived neurotrophic factor), GDNF (glial cell line-derived neurotrophic factor), TGFB-3 (transforming growth factor beta-3), dbcAMP (dibutyryl-cAMP) and ascorbic acid was used. After 4 weeks of neuronal differentiation, derived cells showed positive labeling for early neuronal markers such as TuJ1 and Doublecortin (Figure S4a and b) as well as for the advanced maturation marker MAP2 (Figure S4c). The neuronal differentiation protocol enabled hNSCs to differentiate into GABAergic (GABA, 36.21%), glutamatergic (vGlut1, 40.34%) and dopaminergic neurons (TH, 12.68%) (Figure S4d–f), whereas differentiation into GFAP-positive cells was low (Figure S4g).

To test these results at the mRNA level, we used RT-qPCR to quantitate expression of the neural stem cell marker Nestin, the proliferation marker Ki67, the neuronal marker MAP2, as well as the neuronal sub-type markers for GABAergic (GABA), glutamatergic (GLUT, vGlut1) and dopaminergic (TH) neurons. This analysis was performed in a comparison of hNSC under maintenance conditions and after 4 weeks of neuronal differentiation (Figure S4h). The results demonstrate that neural stem cell identity and proliferation capacities decreased with differentiation. At the same time, we observed increased expression levels of MAP2, GABA, vGlut1 and TH (Figure S4h), indicating ongoing neuronal differentiation.

To examine whether these neurons were functional, we performed immunolabelling for synaptic markers after 4 weeks of differentiation. We reliably detected the pre- and postsynaptic markers synaptophysin and PSD95 (Figure S5a–d and S5e−h), consistent with the expression of a more mature neuronal phenotype. Next, we performed whole-cell patch-clamp recordings following standard procedures. Under voltage clamp conditions, neurons showed a fast transient inward current in response to depolarizing voltage steps (−1.9 ± 0.4 nA, n = 30; Figure S5i) that was identified as a sodium (Na^+^-) current by blocking its influx with 0.5 μM TTX (Figure S5j). The transient Na^+^-current was followed by an outward current with partial inactivation, typical for a mixture of voltage activated potassium channels. This outward current included an A-current that was identified in 10 out of 12 cells tested with a pre-pulse protocol (data not shown). The input resistance of the induced neurons was 1.5 ± 0.2 GΩ (n = 33). The amount of sodium current as well as the input resistance are typical for mature neurons^39^,^40^. In current clamp recordings, cells showed spontaneous action potential (AP) firing, when depolarized to a membrane potential near −50 mV by current injection (Figure S5k). Depolarizing currents elicited APs with +19 ± 5 mV peak potential (n = 15 cells; Figure S5l) and a width at half-maximal amplitude of 3.8 ± 0.1 ms (n = 10). While the generation of APs is an important aspect of the neuronal lineage, we observed no spontaneous postsynaptic currents in continuous voltage clamp recordings (>3 min per cell; n = 30). Similarly, evoked postsynaptic currents could not be elicited by moderate depolarization with KCl applied in bath solution (10 mM; n = 5) or by puffs with high KCl (150 mM; 1–5 s duration; n = 6) directly onto patched cells. In contrast, puff-application of glutamate (1 mM, 0.5 s) elicited inward currents (608 ± 362 pA; n = 4) under voltage clamp conditions (Figure S5m), or depolarizations to membrane potentials near 0 mV (n = 4) during current clamp (Figure S5n). These data indicate the presence of typical sodium channels and glutamate receptors in differentiated hNSCs cells, however, they also indicate that the synaptic release machinery is still incompletely developed, requiring more research into the specific factors regulating its differentiation.

### Transplanted hNSCs survived and differentiated *in vivo*

Finally, we aimed at characterizing hNSC derived cells *in vivo*. The use of hiPSC-derived cells mandates that firstly, transplanted cells are devoid of tumor formation potential, and secondly, the transplanted cells are able to survive *in vivo*. Since the potential of tumor formation is inversely correlated to the degree of differentiation, we selected cells after 6 passages for transplantation because nearly all hNSCs were Nestin-positive and Oct4-/Nanog-negative at this time point. Stereotactic injection of self-renewing hNSCs into the subventricular zone of NOD/SCID mice was never followed by any tumoral growth. In total, 1 × 10^6^ to 2 × 10^6^ hNSCs were transplanted into the brain hemispheres of 9 mice. 3 mice were sacrificed after 6 weeks, 3 other mice were sacrificed after 3 month and the final 3 mice were sacrificed after 6 months. In none of these mice, we observed tumoral outgrowth.

**Figure 7:**
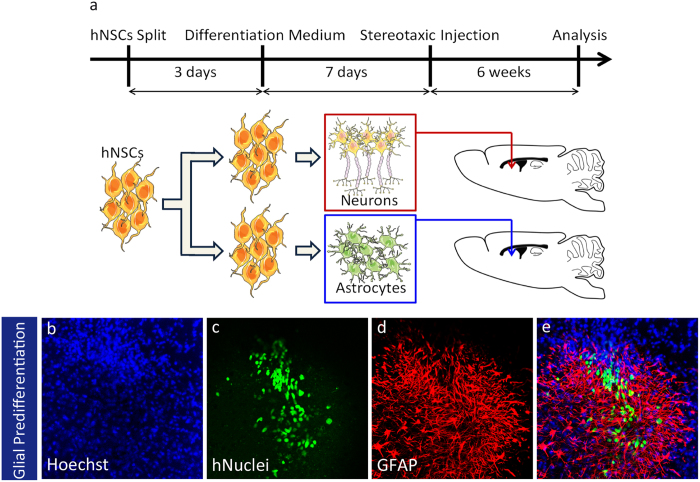
Transplantation of hNSC derived neurons and astrocytes in NOD/SCID mice. (**a**) Schematic representation of the protocol followed. hNSC were kept in maintenance medium for 3 days after splitting. Then the maintenance medium was switched to glial differentiation medium. Stereotactic injection into the subventricular zone of NOD/SCID mice was performed 7 days later. Representative images showing that clusters of GFAP positive cells formed by pre-differentiated hNSC (**b–e**). Drawings were produced using Servier Medical Art (www.servier.com; http://creativecommons.org/licenses/by/3.0/).

To control cells fate after transplantation, hNSC were subjected to a pre-differentiation step. The cell fate was analyzed 6 additional weeks later (Fig. 7a). Since injected cells were of human origin, the transplant could be identified by immunofluorescence with a specific antibody directed against human nuclei (hNuc). Human NSCs that were neuronal pre-differentiated one week before transplantation, were able to survive and to differentiate into TuJ1 (Figure S6c) and Doublecortin (Figure S6g) positive neurons. hNSCs that were differentiated to astroglia for 1 week prior transplantation formed clusters of GFAP positive astrocytes (Fig. 7d).

These data demonstrate that pre-differentiation prior to transplantation can direct the fate of transplanted cells after grafting. The ability to define the fate of cells after transplantation is of outstanding importance for controlled cell replacement therapies. Thus the here described hNSC system may provide a basis for further investigations aiming at the development of strategies for future therapeutic approaches.

## Discussion

In this manuscript, we describe a novel protocol for the generation of hNSCs from hiPSCs. This fate transition is achieved by the chronological administration of media with defined compositions. The here presented protocol is very robust and independent of any sorting method. In contrast to other protocols for the generation of human neural precursor cells, no small molecules were needed for keeping hNSCs under self-renewing condition^12^,^41^,^42^,^43^. Differently to previous studies where neural progenitors grow and proliferate as neurospheres^44^ or neural rosettes^45^, hNSCs were homogeneously maintained in a two-dimensional adherent cell system. Spontaneous differentiation of hNSC into other cell types was negligible. hLIF in the media blocked fate transition of hNSC into neuronal cells. Although hLIF has been reported to induce astrocytes differentiation in synergy with bone morphogenetic protein (BMP) 2 from mouse neuroepithelial cells^46^, we did not detect significant amounts of GFAP-positive cells in the hNSC under maintenance condition. The beneficial effect of LIF has been previously observed in human neural progenitor cells in terms of prevention of apoptosis by inhibition of caspase 3 and 7 47. LIF also provided protection against reactive oxygen species and enhanced cell proliferation^47^. These effects have shown to be driven by the activation of JAK-STAT3 (Janus kinase-Signal transducer and activator of transcription 3) and MEK (MAPK/ERK kinase) pathways in non-neural stem cells such as mouse embryonic stem cells^48^ and human neural progenitor cells^47^.

The here described cells conserved the two main characteristics of neural stem cells, i.e. self-renewing and multi-linear differentiation capacities. Since they grow in homogenous cultures, these cells are an attractive tool for expression profiling, disease modeling and high content screenings. Besides their ability to differentiate into functional neurons, hNSCs differentiated into glial cells within a relatively short time period. In this study, we determined a gene expression profile that distinguishes hNSC from less differentiated hiPSC and more differentiated neurons and astrocytes. Since these profiles were generated from cells from the same individual (starting iPSC line), the degree of comparability is very high and the derived signatures should purely represent the differentiation status.

Several papers described the generation of human astrocytes from fetal or adult post-mortem central nervous system by the expansion of neuronal precursors^49^,^50^. This approach required 6 months to generate a pure population of astrocytes^51^. Other recent papers described the differentiation of astrocytes from iPSC with protocols requiring from 35 days^52^ up to 4 months^53^. Importantly, the obtained populations seem to represent astrocytes just in a reactive form as shown by the almost 100% immunoreactivity for GFAP. Therefore, these cultures might not be suitable to completely model mature astrocyte functions or to mirror patho- and physiological conditions^33^.

In the here presented study, the derivation of astrocytes was achieved by a cost-efficient media composition, which ensures a highly pure culture as shown by the negligible contamination with Tuj1 positive cells. Unlike other protocols^54^, our protocol is simple and does not require any antibody-based sorting step of glia or neuronal progenitors. Remarkably, we were able to obtain a population of mature astrocytes both in a quiescent state with a protoplasmic morphology (negative for GFAP) as well as in a reactive phenotype characterized by GFAP expression. The expression of EAAT2 in all the cells and the ability to uptake glutamate strongly supported the acquisition of mature functions. The importance of this feature was highlighted by the different effects of immature and mature astrocytes on axonal regeneration^55^,^56^.

The high pyruvate carboxylase activity confirmed the acquisition of metabolic specialization of hNSC-derived astrocytes as pyruvate carboxylation is an important anaplerotic reaction, specifically occurring in astrocytes^57^,^58^. Moreover, the relative glucose flux into the TCA cycle was higher in astrocytes resulting in higher glucose derived carbon contribution to glutamate^59^. Very interestingly, in astrocytes as well as MLDCs around 50% of the citrate carbons derived from other sources than glucose or glutamine. These other sources might be represented by lipid oxidation and/or degradation of amino acids such as branched chain amino acids. Compared to other cell lines 50% of carbons derived from alternative carbon sources is relatively high, at least at basic culture conditions and high oxygen tension (unpublished data). The reasons for that can be multiple and should be investigated in future research. Identifying the relevance of alternative carbon sources for glial specific metabolism could help for a better understanding of cell survival and integrity.

Particularly interesting is the role of serine as it represents an important metabolic intersection point to i) provide one-carbon units to the folate mediated one-carbon metabolism, to ii) serve as a precursor for the transsulfuration pathway to generate cysteine from serine and homocysteine and iii) as the precursor to produce the non-essential amino acid glycine. Serine and its connected metabolic pathways have been shown to be of special importance in neuronal oxidative stress conditions^60^. Astrocytes have an important supportive function in protecting neurons against oxidative stress by providing the antioxidant glutathione^61^. Glycine represents together with cysteine and glutamate one of the three amino acids that build the antioxidant glutathione.

Recently, the outstanding importance of astrocytes for neurological disease got into focus of several research approaches. As an example, it has been shown that astrocytes strongly contribute to the development of the Down syndrome^62^. Additionally, a recent study clearly demonstrated that transplantation of astrocytes was extremely beneficial in a rat model of Parkinson’s disease^63^. Thus, the availability of an effective method to generate mature astrocytes, as described in this study, is of key importance for convincing disease-modelling studies and replacement therapy strategies. This is becoming a relevant field of investigation especially for neurodegenerative diseases such as Parkinson´s and Alzheimer´s disease.

## Additional Information

**How to cite this article**: Palm, T. *et al.* Rapid and robust generation of long-term self-renewing human neural stem cells with the ability to generate mature astroglia. *Sci. Rep.* **5**, 16321; doi: 10.1038/srep16321 (2015).

## Supplementary Material

Supplementary Information

Supplementary table 1
