# Supplementary material for: Rapid and robust generation of long-term self-renewing human neural stem cells with the ability to generate mature astroglia
Source: Sci Rep. 2015 Nov 6;5:16321. doi: 10.1038/srep16321 (PMC4635383; doi:10.1038/srep16321)
Supplement: Supplementary Information [file srep16321-s1.pdf]

Rapid and robust generation of long-term self-renewing human neural stem cells with the ability to generate mature astroglia

- Supplementary Information –

Thomas Palm[1*], Silvia Bolognin[2*], Johannes Meiser[2], Sarah Nickels[2], Claudia Träger[1], Ralf-Leslie Meilenbrock[1], Johannes Brockhaus[3], Miriam Schreitmüller[3], Markus Missler[3,4] and Jens Christian Schwamborn[1, 2]

*Equal contribution

[1] Stem Cell Biology and Regeneration Group, Institute of Cell Biology (ZMBE), Westfälische Wilhelms-Universität Münster, 48149 Münster, Germany

[2] Luxembourg Centre for Systems Biomedicine (LCSB), University of Luxembourg, Esch-Belval, Luxembourg

[3] Institute of Anatomy and Molecular Neurobiology, Westfälische-Wilhelms University, Münster, Germany

[4] Cluster of Excellence EXC 1003, Cells in Motion, CiM, Münster, Germany

Running Title: Generation and characterization of hNSCs and glia

Supplementary Figure Legends

Figure S1. Multilinear differentiation of hNSCs

Multilinear differentiation is achieved by culturing hNSCs in the basic medium, supplemented with 10% of FCS. Stem cells differentiated into TuJ1 positive neurons (a) and GFAP positive astrocytes (b).

Figure S2. Gene expression profiling of hNSCs

(a) Growth curve of hNSCs maintained under proliferative conditions over 21 Passages. (b) Paired analysis of the microarray based expression intensities of hiPSC and hNSCs associated genes.

Figure S3. Differential gene profiling between hiPSCs, hNSCs, and hMLDCs.

Transcriptional analysis and heat map correlation showing 2587 transcripts differentially regulated in hiPSCs, hNSCs and hMLDCs.

Figure S4. Derivation of functional neurons.

(a-f) Representative confocal images illustrating the expression of neuronal markers Tuj1 (a), DCX (b), MAP2(c), GABA-Tuj1 (d), vGlut1-Tuj1 (e) and TH-Tuj1 (f). (g) Quantification of the number of neurons positive for MAP2, Tuj1, vGLUT1, GABA, TH, GFAP. (h) mRNA expression levels of *Nestin, Ki67, MAP2, TH, GABA* and *GLUT1* in hNSC and neurons quantified by RT-qPCR.

Figure S5. hNSCs derived neurons develop synapses and electrophysiological activity.

(a-h) Representative confocal images illustrating the expression of neuronal markers synaptophysin (j), NCAM (k), PSD95 (n), Tuj1 (o). (i) Current-voltage protocol (-60 to +20 mV, 50 ms) shows fast inward current and slower outward currents. (j) The fast inward current is identified as Na^{+}-current by its sensitivity to TTX (0.5 µM; red trace). (k) In current clamp recordings cells fired spontaneous action potentials (APs), when the membrane potential was set to -50 mV. (l) Current steps (5 - 15 pA, 0.3 s) activated overshooting APs, stronger injections elicited series of APs with moderately declining amplitudes. (m) Puff-application of glutamate (1 mM) from a glas pipette (15 µm tipp diameter) placed 50 - 80 µm away from the recorded cell elicited an inward current when the cell was voltage clamped to -70 mV. (n) During current clamp, the cells depolarized in response to the glutamate puff.

Figure S6. Transplantation of hNSC derived neurons in NOD/SCID mice.

(a-h) Representative images of the subventricular zone showing that the pre-differentiated hNSC differentiate into Tuj1 (c) and Doublecortin (DCX) (g) positive cells.

Table S1. mRNA differentially expressed upon differentiation

List of mRNA differentially expressed between hiPSCs (group 1), hNSCs (group 2), and hMLDCs (group 3). Statistical analysis was performed using ANOVA followed by Bonferroni´s multiple comparison test.

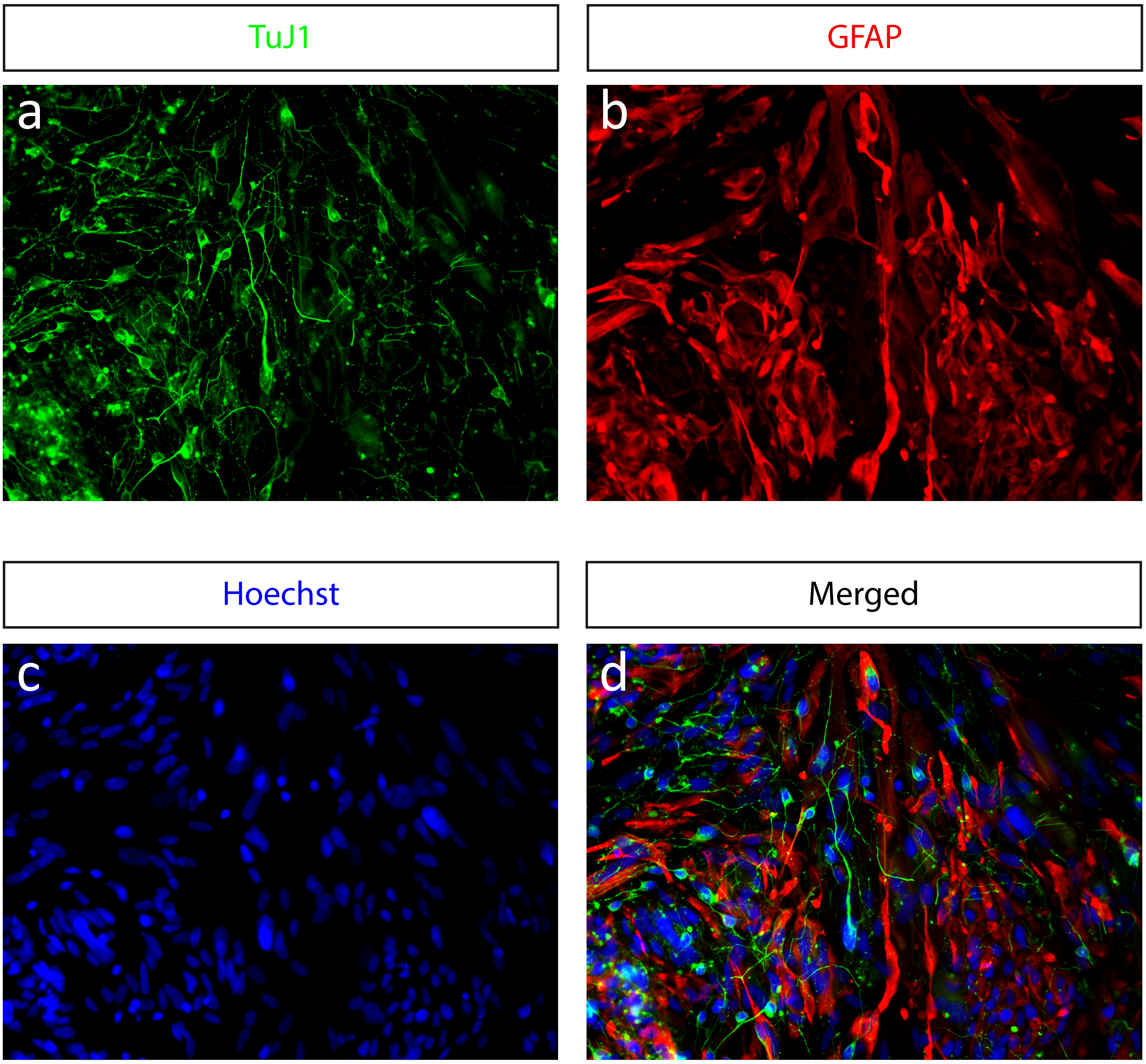

Figure S1

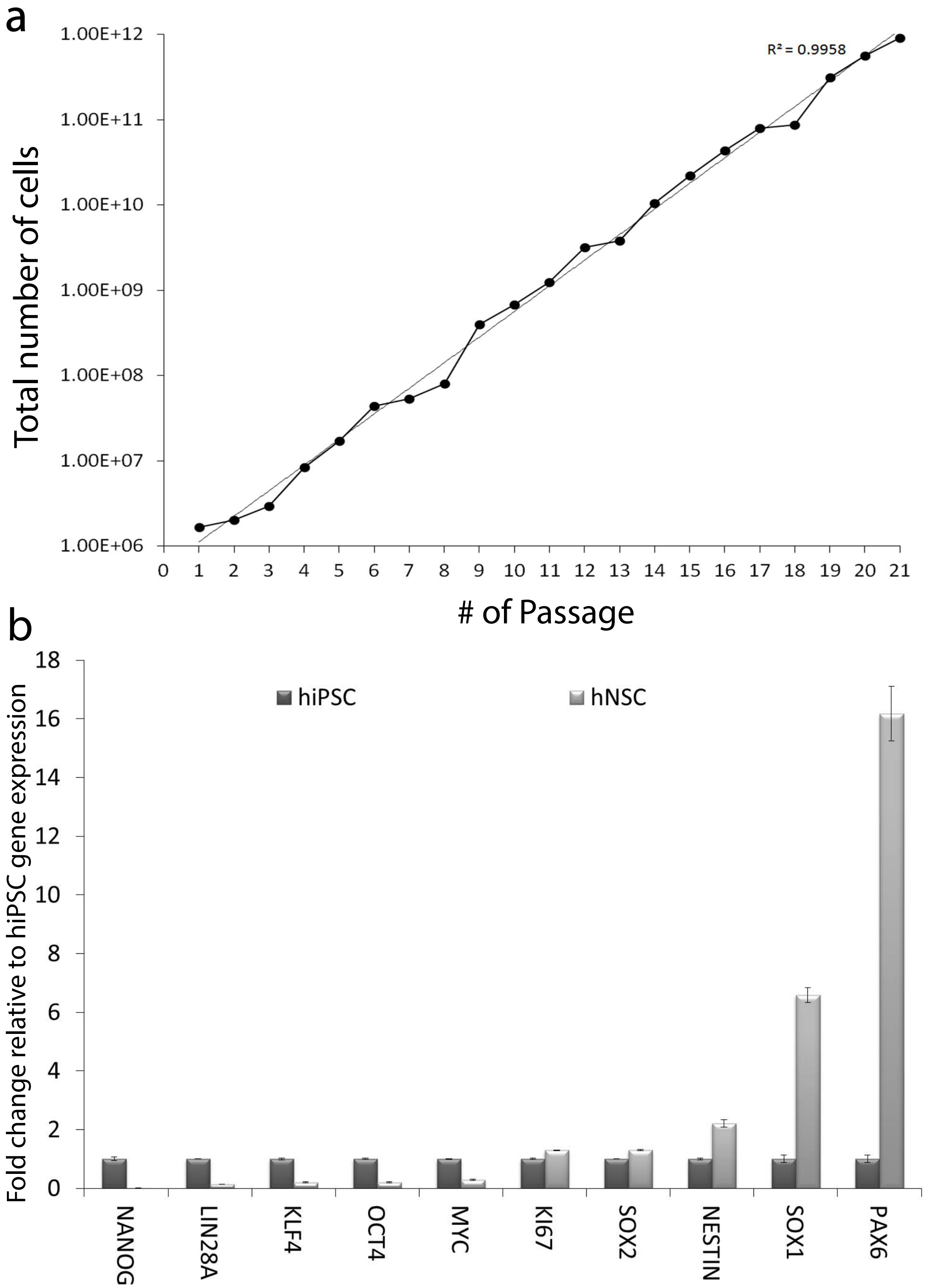
a
R² = 0.9958
Total number of cells
1.00E+12
1.00E+11
1.00E+10
1.00E+09
1.00E+08
1.00E+07
1.00E+06
0 1 2 3 4 5 6 7 8 9 10 11 12 13 14 15 16 17 18 19 20 21
# of Passage
b
Fold change relative to hiPSC gene expression
18
16
14
12
10
8
6
4
2
0
hiPSC
hNSC
NANOG
LIN28A
KLF4
OCT4
MYC
KI67
SOX2
NESTIN
SOX1
PAX6

Figure S2

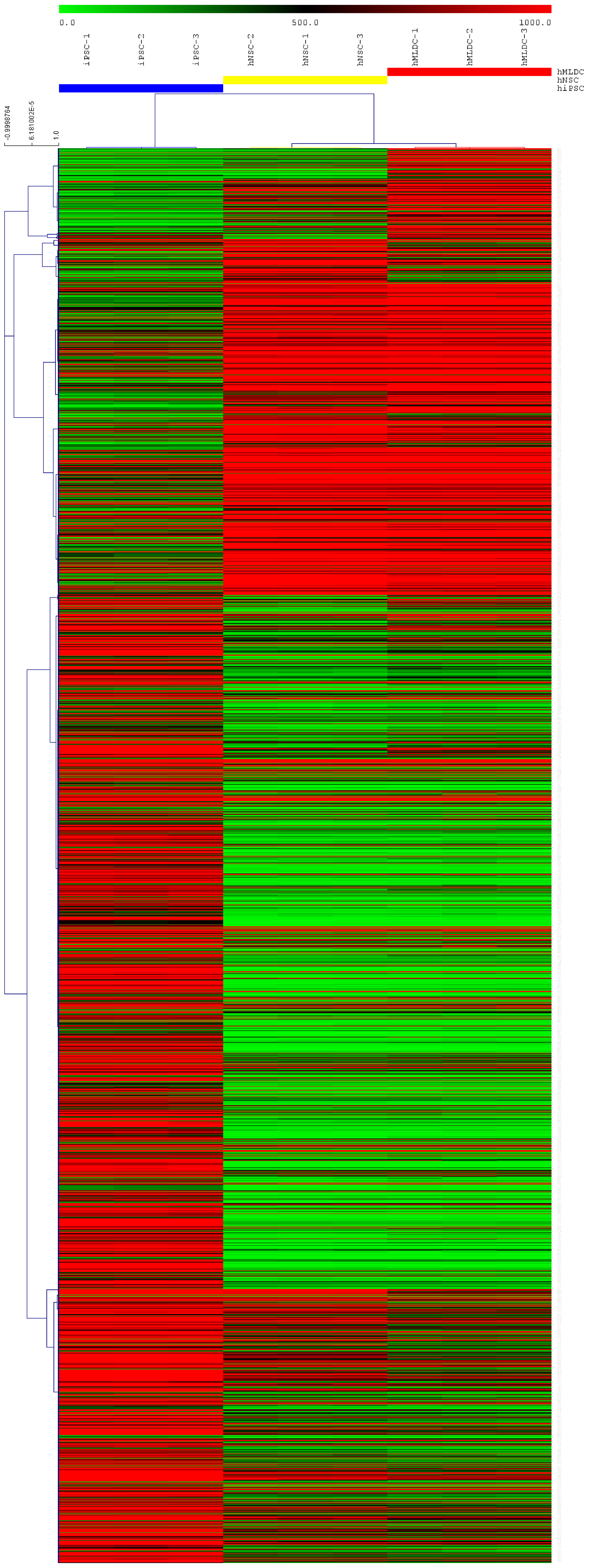
0.0
500.0
1000.0
iPSC-1
iPSC-2
iPSC-3
hNSC-2
hNSC-1
hNSC-3
hMLDC-1
hMLDC-2
hMLDC-3
hMLDC
hNSC
hiPSC
-0.9998764
6.181002E-5
1.0

Figure S3

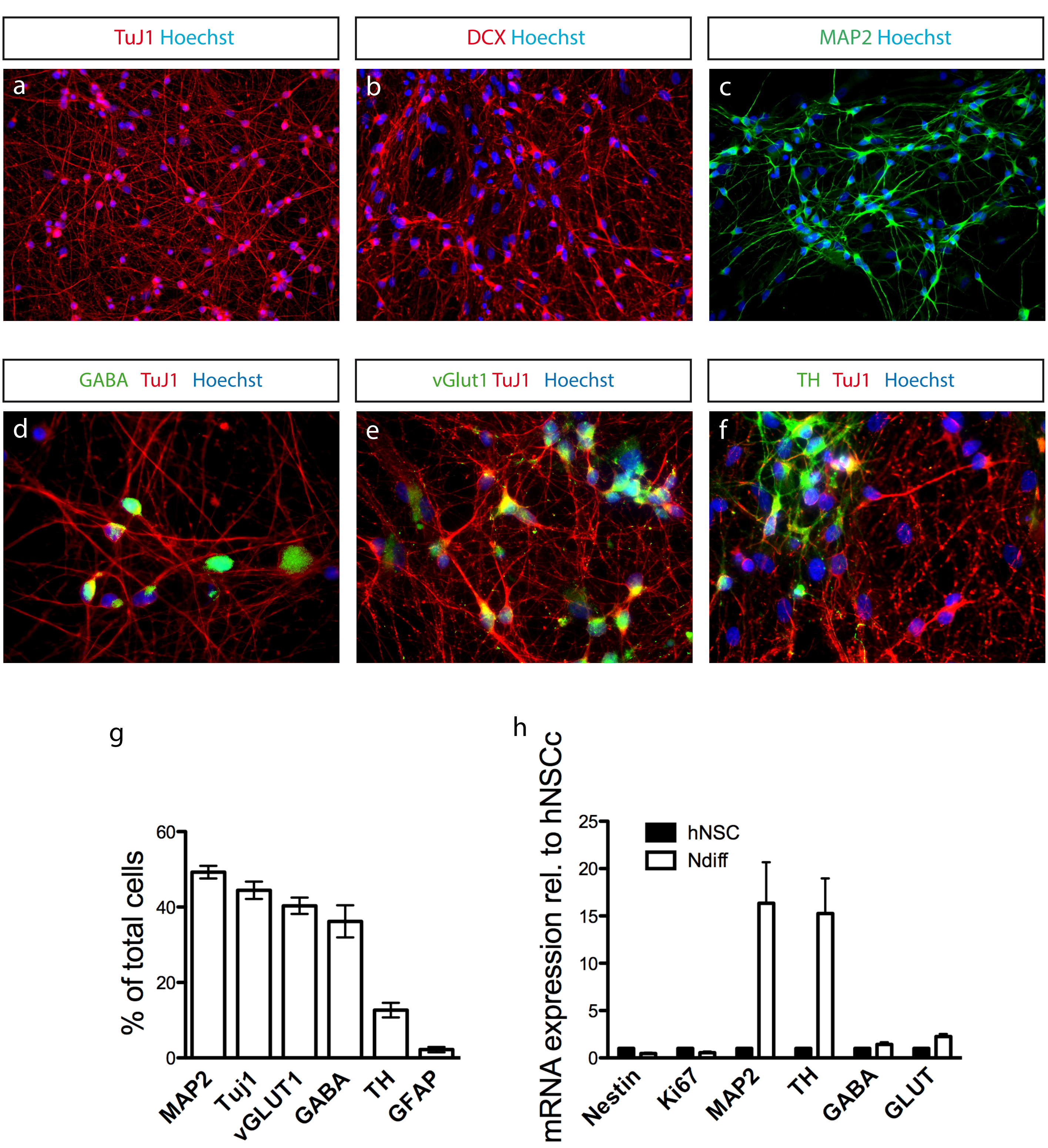
TuJ1 Hoechst
DCX Hoechst
MAP2 Hoechst
a
b
c
GABA TuJ1 Hoechst
vGlut1 TuJ1 Hoechst
TH TuJ1 Hoechst
d
e
f
g
% of total cells
60
40
20
0
MAP2
Tuj1
vGLUT1
GABA
TH
GFAP
h
mRNA expression rel. to hNSCc
25
20
15
10
5
0
hNSC
Ndiff
Nestin
Ki67
MAP2
TH
GABA
GLUT

Figure S4

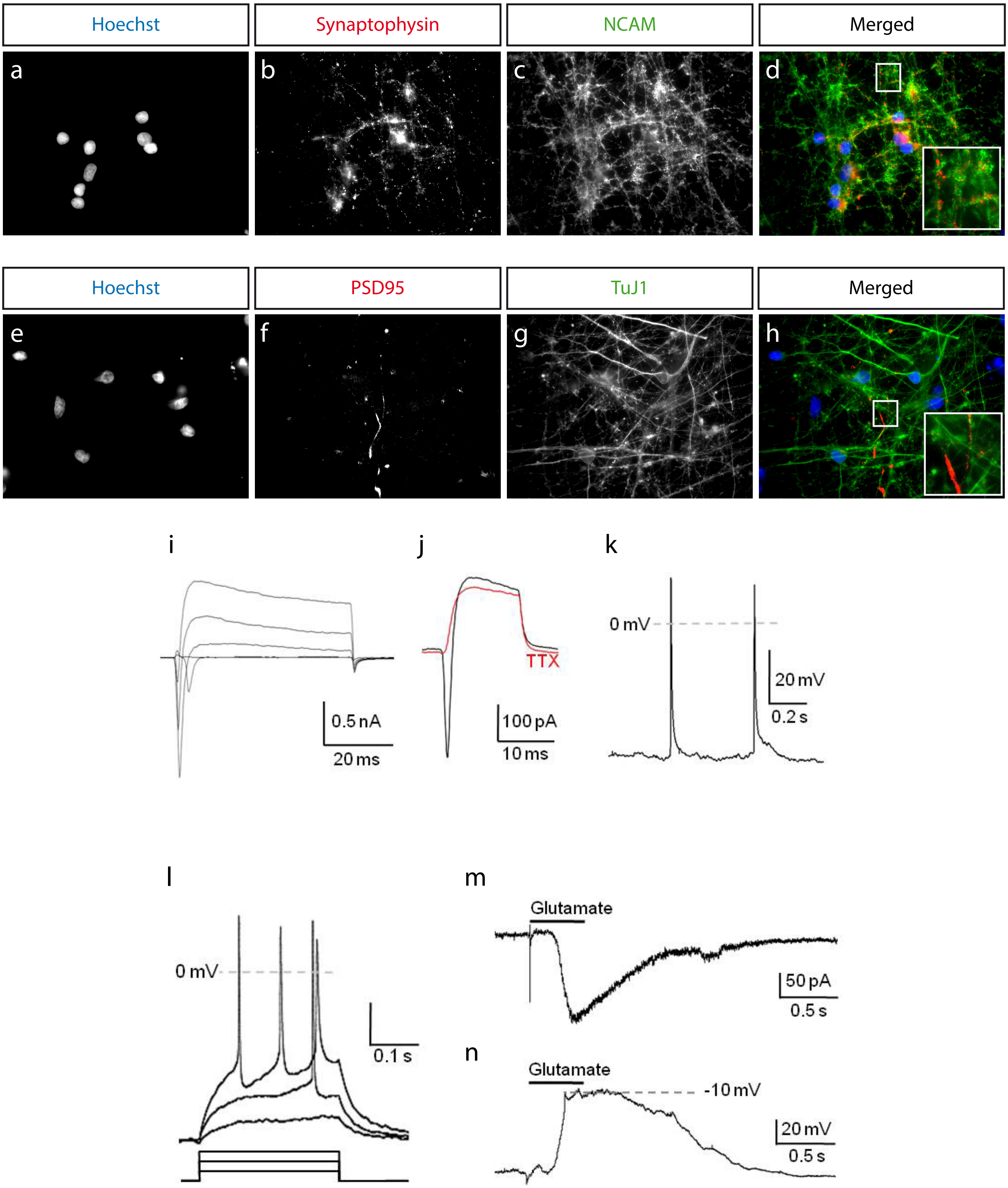

Hoechst
Synaptophysin
NCAM
Merged
a
b
c
d
Hoechst
PSD95
TuJ1
Merged
e
f
g
h
i
0.5 nA
20 ms
j
TTX
100 pA
10 ms
k
0 mV
20 mV
0.2 s
l
0 mV
0.1 s
m
Glutamate
50 pA
0.5 s
n
Glutamate
-10 mV
20 mV
0.5 s

Figure S5

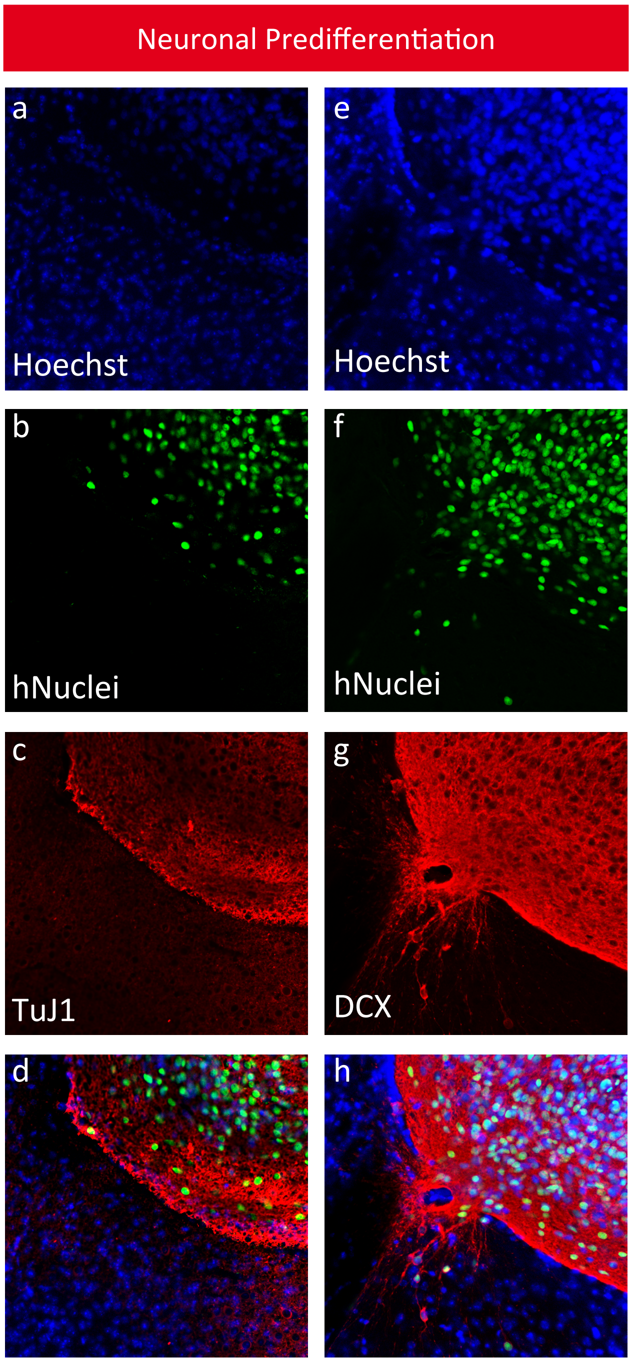
Neuronal Predifferentiation
a
Hoechst
e
Hoechst
b
hNuclei
f
hNuclei
c
TuJ1
g
DCX
d
h

Figure S6
